# A short guide to addressing accessibility at scientific conferences

**DOI:** 10.1242/jcs.261858

**Published:** 2024-05-24

**Authors:** Urszula Lucja McClurg

**Affiliations:** Institute of Systems, Molecular and Integrative Biology, University of Liverpool, Biosciences Building, Crown Street, Liverpool L69 7ZB, UK

**Keywords:** Accessibility, Conference inclusivity, Disability

## Abstract

Although encouraging progress to address issues of accessibility at scientific conferences has been made in recent years, further efforts are required to enact the comprehensive solutions necessary to accommodate the diverse needs of disabled scientists. This Opinion provides an easy-to-follow guide to ensuring that scientific conferences are accessible to disabled scientists and is aimed at conference organizers and funders in the field of cell biology. In this piece, I, a person who identifies as a disabled scientist, advocate for collective action within the cell biology community to promote the routine inclusion of accessibility officers on conference organizing panels and the use of accessibility checklists as part of applications for conference funding in order to build inclusive practices into conference planning and organization. I propose a move away from requiring personal disclosures of disability needs on a person-to-person basis towards community-agreed guidelines that ensure accessibility for scientists with a wide variety of needs. To that end, I detail a list of practical, cost-effective adjustments to standard conference activities that can enhance accessibility. Moreover, I suggest several long-term, high-impact changes – including guaranteeing the availability of wheelchair-accessible facilities and making hybrid meeting formats standard – aimed at enabling conference participation for all scientists.

## Teaser

**Summary:** Solutions to improve the accessibility of scientific conferences are proposed, including designated accessibility officers, accessibility checklists and measures to avoid personal disclosures of disability.

## Introduction

Recently, the cell biology community has engaged in increasing efforts to improve the accessibility and inclusivity of scientific conferences. Initiatives such as the NewPI in Cell Development Forum, which started during the COVID-19 pandemic, are paving the way for innovation in conference accessibility, inclusion and sustainability. The first NewPI in Cell Development conference was held in Denmark in 2022 and provided a platform to discuss exciting solutions to challenges in conference sustainability and inclusion (Chalmers et al., 2023). Similarly, the Dynamic Cell meetings (https://www.eventsforce.net/biochemsoc/frontend/reg/thome.csp?pageID=75474&eventID=151) organized jointly by the British Society for Cell Biology and the Biochemical Society have been making strides in improving accessibility, provisions for attendees observing religious practices such as fasting during Ramadan and inclusion of resources for early career researchers within our field. These initiatives show that there is a great deal of good will in our community towards increased inclusion. However, one of the communities that often falls through the cracks of conference accommodations is the disabled community.

To move forward with improving conference accessibility for the disabled community, it is important to understand where past and current barriers to conference accessibility for disabled scientists originate. First, there is no single formula for evaluating disability, nor is there one magic solution to ensuring accessibility. On the contrary, there are as many individual accessibility needs as there are disabled scientists. Even within the disabled community, it is difficult to speak for anyone else but oneself. It is therefore very challenging to devise a list of changes and initiatives for improving conference accessibility that will address every diverse condition and circumstance. Thus, we will only be able to develop consensus guidelines by enabling networks of disabled scientists to come together with organizing panels in order to consider improvements to conference accessibility.

Second, the other major barrier to accessibility is the tendency to want to stick to the status quo. As much as we aim to be innovative in our scientific thinking, institutional structures within academic science – such as publishing, grant review and tenure decisions – have proven resistant to change, showing how much we like to hold on to the system we know (Luebber et al., 2023). Consequently, accessibility requests might be dismissed as unreasonable if conferences are judged to have been successful without making accommodations in the past. This issue is compounded by the complex nature of scientific hierarchy, which prevents disabled scientists from seeking accommodations out of concerns about adverse effects on their careers. Thus, it is essential to address how the expectation that disabled scientists must disclose their conditions in order to seek accommodations discourages and complicates inclusion efforts. In this Opinion, I discuss how the status quo of conference organizing hinders accessibility and present a short guide to approaches that organizers can take to ensure inclusion of disabled scientists within the cell biology community and beyond.

## Accessibility shouldn't be a privilege reserved for more established scientists

One suggestion often made to address conference accessibility is to ask attendees to disclose their specific needs so that they can be accommodated on a case-by-case, meeting-by-meeting basis. This tendency perhaps stems from the flawed assumptions that disabled scientists are extremely rare, which is not the case (according to a 2021 report from the National Science Foundation, 5.0% of individuals with doctorates in science, engineering or medicine under the age of 40 and 9.4% of those aged 40 or over have a disability; https://ncses.nsf.gov/pubs/nsf21321/), and that accessibility needs are so individualized that they cannot be anticipated. However, details of any individual's medical history are classified information reserved for medical professionals, and demanding disclosures prior to considering accessibility requests is highly inappropriate. Furthermore, some disabilities may be distressing for the individual to disclose and discuss. For example, someone with irritable bowel syndrome may be unwilling to explain to a leader in their field why they need accommodations involving frequent breaks during sessions or certain seating arrangements. Similarly, a PhD student with endometriosis might not feel comfortable talking to a senior male PI about accommodations to help with period needs and pain. These examples hopefully help to explain why expecting personal disclosures as a rule is not acceptable. Furthermore, placing the onus for arranging accommodations on disabled attendees sends a message that it is difficult and disruptive to offer accommodations to those who need them. This ultimately perpetuates the impression of disability being unwelcome in the scientific community.

It is also important to remember that conferences are professional events. By law, in most countries, individuals have the right not to disclose their disability during hiring and interview processes in order to protect them from employment or workplace discrimination. By demanding that attendees disclose their disability and accessibility needs to conference organizers, we are breaching this legal line that protects against discrimination. Many communities specific to different scientific fields are small, and information disseminates quickly within them. It is important to acknowledge that conference organizers are not medical professionals under legal obligation of confidentiality. Inappropriate discussion of disclosures could thus impact attendees’ future job prospects, or at the very least leave them with the uncertainty of whether their disclosure of accessibility needs was a deciding factor in subsequent employment decisions. Therefore, I strongly urge conference organizers to move away from requiring personal disclosures from attendees and adopt consensus guidelines that ensure accessibility is built into conferences at the outset. Next, I present a series of such guidelines that are designed for the community to consider and build upon.

## What should we change?

The guidelines detailed below are not intended as a comprehensive and complete list of proposed changes, but as a collection of improvements in various areas of accessibility that are based on the author's own experiences of attending and organizing conferences, as well as suggestions gathered by speaking to other disabled academics. These recommendations are categorized into those that simply involve a change of mindset, are often cost free and could be implemented without delay, versus large-scale changes that will require investment of time and resources.

### Simple, low-cost changes

#### Accessibility officers ensure inclusion from conference planning to execution

To promote inclusivity, it is important to ensure that conference organizing panels are diverse and represent the interests of different scientists (e.g. scientists who are disabled, marginalized, of different nationalities and at various career stages) (Joo et al., 2022). It is relatively easy to adjust plans in the early phases of conference organization; however, once all the decisions about location and catering have been made, accommodating previously unanticipated accessibility needs becomes complicated. For this reason, it is important to ensure that accessibility needs are addressed from the outset of planning. I propose that this can be achieved by designating an accessibility officer on the organizing panel of every conference. The role of an accessibility officer should involve advocating for accessibility during conference planning and organization, as well as highlighting any inaccessible aspects of planning. This person should be familiar with disability and have completed accessibility needs training. Furthermore, during the conference the accessibility officer should be the point of contact for any unforeseen accessibility needs. The officer should be highlighted in the programme, their phone number should be made available to all attendees, and they should wear a colour-coded badge that is visible at all times so that everyone at the conference can easily identify them. They should ideally be local to the venue, or at a minimum speak the language of the country where the conference is held, so that they can support attendees in case of emergencies. The accessibility officer should also be involved in post-event reporting on conference success and outcomes. Many conferences are a part of a recurring series, and seeking feedback from attendees with specific questions around accommodations and accessibility will ensure that meetings improve with each instalment.

#### Knowledge is power for attendees with accessibility needs

At the time of registration, conference programmes tend to include only general information about the conference venue and session timings. However, conference attendees are usually not familiar with the venue where the meeting will take place and cannot easily inspect it in advance. For many people with accessibility needs, planning how to get to and navigate a meeting requires specific knowledge of venue details, such as the floor that the talks will be held on, the availability of an elevator, the number of steps that will need to be taken and the distance from the meeting room to the toilets. These critical details can make the difference between an individual being able to attend a meeting or not. Conference organizers should therefore provide detailed information about the venue, hotel(s) and transportation to the venue in the programme ahead of the registration deadline to allow disabled scientists to judge whether accessibility will be an issue and enable them to plan appropriately.

#### Avoid room sharing by default to prevent forced disclosures

Conferences frequently require attendees to share hotel rooms. However, this can put disabled scientists in a compromising situation. First, some disabilities might cause the attendee to feel embarrassed. As a consequence, forcing them to share a room with a stranger might cause genuine distress. Second, even though some disabilities are described as ‘invisible’, very few remain invisible in the context of sharing a hotel room. Hiding symptoms or use of medical equipment and medication is often impossible and should never be asked of anyone. As mentioned above, disabilities fall under protected medical information, and disclosure of such information should never be forced in a professional setting. Room sharing by default should thus never occur at conferences and should only happen when specifically chosen and requested by individual attendees.

#### Send a message that wheelchair users are welcome

Designated spaces for wheelchairs should be indicated in seminar rooms. Attending a conference usually requires wheelchair users to navigate tightly packed seminar rooms filled with chairs that then need to be moved out of the way by other attendees. To send the message that wheelchair users are welcomed and planned for at a conference, clearly marked spaces reserved for a wheelchair in an easily accessible location are essential.

#### Microphone use enables participation of those with hearing aids

One of the most critical factors for attendees at a conference is being able to hear the speakers. Some of us rely on hearing aids for this. Hearing aid users can struggle with hearing speakers in conference rooms, which are often large spaces with tall ceilings where noise echoes. Many speakers might not be aware of this and choose not to use a microphone, even if one is provided, if they think that their voice projects enough. Furthermore, some hearing aids rely on being coupled with the microphone channel in the seminar room. It is important that conference chairs and moderators are aware of this and that nothing is communicated without the use of microphones, including informal question and answer (Q&A) sessions following presentations. Conference venues that have rooms where hearing aids can be coupled with the microphone input should thus be selected for conference sessions.

#### Slide formatting assists those with visual sensitivities

Conference presentation slides can be difficult to process for attendees with conditions such as dyslexia, dyspraxia or attention deficit hyperactivity disorder (ADHD). Some people with dyslexia find it difficult to absorb information on slides without sufficient contrast and can rely on screen sheets or colour overlays, which reduce visual stress, to interpret material in their daily life. The ideal slides for attendees with visual sensitivities should have dark text on a light (but not white) background (Mitchell et al., 2008). To improve accessibility of presentations, conference organizers should include slide guidelines that encourage dyslexia-friendly formatting when inviting speakers.

#### Timekeeping is everything for those on medication or with complex needs

Conferences tend to have detailed schedules that are planned out to the minute with carefully organized short breaks. However, speakers often overrun their allotted presentation time, and schedules can slip as the meeting goes on. This can cause complications for many disabled attendees. For example, those on strict medication regimes, for whom taking medication is often not as easy as just swallowing a pill, might rely on breaks occurring strictly as scheduled in the programme. This is also true for those who rely on medical equipment that needs dedicated time for cleaning or servicing. Thus, clear and reliable information about break times is used by disabled attendees to plan medical routines. Consequently, timekeeping needs to be adhered to during the conference. Session chairs need to be made aware of the implications of moving break times by allowing sessions to overrun and need to work more proactively to ensure that timetables are respected.

#### Allotting extra time aids speakers with speech impediments

Adherence to specific pre-set timings for presentations can become discriminatory when there are speakers with speech impediments such as a stutter. During conference planning, once all speaking slots are assigned, speakers should have the opportunity to request 10% additional speaking time if they have a speech impediment that affects their ability to keep to the allocated presentation time. This would not represent a request for self-disclosure, as such impediments would be invariably disclosed to the organizers and audience during the talk. It is also critical that these changes are made before the programme is released to the attendees, so that timekeeping can be maintained to support those that need to plan medication use and healthcare around break times. Additionally, if speech impediments are exasperated under stressful conditions, speakers should be allowed to submit a prerecorded version of their talk that can be combined with a live Q&A session.

#### Not all of us can stand it – providing seating during poster sessions

Poster sessions are in general a positive element of conference programmes and offer a great opportunity for junior scientists to disseminate their work and build their CVs. However, poster sessions also often require presenters to stand on the spot for long periods of time, typically up to several hours. Furthermore, attendees interested in browsing posters often find themselves in small, cramped spaces without a place to sit. Many invisible disabilities, such as fibromyalgia, endometriosis and Ehlers–Danlos syndrome, make it incredibly challenging for individuals to stand for a prolonged period of time (Hakim et al., 2017; Mesci et al., 2023; Ramin-Wright et al., 2018). Poster sessions should thus be planned so that seating for poster presenters is made available. Furthermore, spaces between posters should be wide enough to allow for easy movement, even for those using walking aids or wheelchairs. Additional chairs should also be provided throughout the poster area for remaining attendees who might also not be able to stand or walk for prolonged periods of time.

#### Make food for those with dietary restrictions available in abundance

Awareness of and planning for allergies, food intolerances and dietary restrictions associated with medication play a vital role in conference accessibility. Organizers should select venues with demonstrated experience in allergy-compatible catering. Provisions should be made for gluten-, dairy- and lactose-free food, and all food should be labelled in a detailed manner. Although information about allergies and dietary restrictions is often requested from attendees upon registration, food compatible with these different restrictions should be provided in greater amounts than requested at that stage. This is because food intended for those with restricted dietary needs is often accidentally taken by attendees without restrictions who simply felt it was the most attractive option at the buffet. For example, if only one gluten-free meal intended for an individual with coeliac disease is made available but is consumed by someone else, this can put the attendee with coeliac disease in the incredibly difficult situation of choosing between consuming gluten, which is very dangerous for their long-term health, or not eating at all. The purpose of ensuring true and carefully considered accessibility is to avoid ‘othering’ those with accessibility needs in this way. Meals that accommodate allergies and food intolerances should therefore be part of the standard spread and not specifically designated only if an attendee requests them.

#### Make sure that water is always an option

At coffee breaks and meals, it is crucial to always make water and alternative beverages such as sugar-free juices, caffeine-free tea and coffee, and gluten-free alcoholic drinks available. Not everyone can have tea and coffee. Caffeine is a muscle stimulant, and people with certain conditions like endometriosis might risk a flare-up when consuming it (Harte et al., 2012); diabetic attendees will require sugar-free drinks; and people who are pregnant, alcohol intolerant, or do not consume alcohol for religious or other personal reasons need non-alcoholic options. Overall, water is the easy and ideal alternative for anyone who might have restricted drinking patterns.

### Big, high-investment changes

#### Choose fully wheelchair-accessible venues

Going forward, accessibility needs to be considered at the heart of conference organizing. Therefore, in the future, only venues that are fully wheelchair accessible should be selected. True wheelchair accessibility means more than just the ability of wheelchair users to access the seminar room. Elevators need to be wheelchair accessible with buttons that can be reached from the wheelchair, doors (especially bathroom doors) must have power-assisted mechanisms to allow access for electric wheelchair users, spaces within the audience that are open for wheelchairs need to be designated, and ramp access to platforms or stages must be provided for speakers who are wheelchair users. Furthermore, conference accommodation should include at least two wheelchair-accessible rooms on the ground floor or with a fully accessible lift and power-assisted doors. This information should be outlined clearly on the conference website before the registration deadline in a separate tab or page dedicated to accessibility. This will allow scientists with accessibility needs to make an informed decision about attending.

#### Ensure accessible travel to the conference venue

Difficulties involving travel to the conference location are a significant factor that renders conferences inaccessible to disabled scientists. Conferences are frequently organized in remote countries or at holiday resorts that are far from an airport or any large city. It is therefore important, not only for environmental reasons, to consider how many connecting flights attendees might require and what modes of transportation are available to reach the conference venue from the airport. Long or multiple flights can be a challenge for anyone with restricted mobility as well as for those who should not sit for prolonged periods of time; for example, attendees with increased risk of deep-vein thrombosis or stroke. Conferences should be held in places that can be easily reached from most academic centres and where transport from the airport to the venue is direct, straightforward and wheelchair accessible.

#### Guarantee private bathroom settings

As mentioned above, many disabilities can create a feeling of embarrassment in certain situations. These embarrassing situations can be avoided by making more private bathroom settings available. This is essential, especially in situations where attendees need to wash and change medical equipment, such as stoma fittings, during the day. It is important for such attendees to have access to bathrooms where the sink is contained within the cubicle, so that this does not need to be done in public. Separate, accessible bathrooms that are self-contained and allow more privacy should be identified, and this information should be provided to attendees. Ensuring access to a sufficient number of bathrooms that are not part of a major facility and preventing long lines and wait times for the bathroom will also reduce stress and pressure on attendees.

#### Provide captions and screen reader support for deaf or blind scientists

As discussed above, for those with hearing difficulties that can be supplemented by a hearing aid, conference attendance can be highly effective when microphones and hearing aid connections are made available. However, this provision is not sufficient for deaf or blind scientists. For that reason, all talks should be made available with captions, for deaf scientists, and with screen reader support, for blind scientists. Slides should be made compatible with captioning and screen reader systems, and presentation slides should not be provided in PDF format, as screen readers cannot read PDF files. Importantly, these provisions will also benefit scientists who might not be confident English speakers, further improving conference accessibility for additional participants.

#### Embrace hybrid meetings

Even when all these provisions are made, it might not always be possible for everyone to attend a conference. People who are currently going through flare-ups or active treatment regimens, or wheelchair users who are worried about damage to their chair caused by air travel, might ultimately opt not to travel. These often-recurring barriers to conference attendance cause obvious detriment to scientific careers and development, which is unjust. Consequently, going forward, all meetings should be organized in a hybrid format. This format allows remote attendees to do more than just access talks via live streaming or post-hoc recordings, enabling them to actively participate in Q&A sessions and workshops. This can be achieved through the creation of hub sites that allow remote attendees to not only listen to talks but also take part in the networking and community-building benefits of conferences (Chalmers et al., 2023). Beyond those with disabilities, hybrid meetings additionally benefit scientists from developing nations who might not have the funds to attend international conferences in person, as well as those who are disadvantaged by immigration policies and are not able to secure visas for travel to the meeting (Wu et al., 2022).

## Conclusions

Accessibility needs and disability status are not set in stone. Many of us will experience increased accessibility needs with ageing as well as intermittent periods of increased accessibility needs following accidents or illnesses, or during and following pregnancy. Improving conference accessibility will therefore benefit everyone in the scientific community, not just those who are disabled today. The list of recommendations provided here is only a starting point. Conference organizers such as Gordon Research Conferences (GRC), the Federation of American Societies for Experimental Biology (FASEB), the European Molecular Biology Organization (EMBO) and other national scientific societies in the cell biology community should actively seek opinions and advice from disabled academics to broaden these recommendations and ensure that all scientists can access their conferences. As these groups benefit financially from organizing conferences, efforts to improve conference accessibility should not be expected to be an uncompensated service provided by marginalized scientists, but should instead be remunerated in accordance with an advisory role. Furthermore, these recommendations should be shared within the greater scientific community to improve conferences worldwide. Organizations that sponsor and endorse conferences should have an accessibility checklist to provide to a designated conference accessibility officer, and meetings should be supported by these organizations only if accessible conditions are ensured. The collective barriers to conference accessibility discussed here are one of the reasons why so few disabled academics become conference speakers. Conferences are where we present our work and build networks and collaborations. Without these opportunities, it is challenging for marginalized scientists to progress in their academic careers. Furthermore, the absence of visibly disabled speakers or attendees from conferences makes it hard for trainees who identify as disabled to imagine themselves succeeding in this profession. By increasing the accessibility of conferences to a more diverse pool of scientific talent, we further the potential to increase diversity in science and extend the reach of our science to more diverse trainees and institutions.
